# A case of one-lung ventilation using a single-lumen tube placed under fiberoptic bronchoscopic guidance in a 4-year-old child: A case report

**DOI:** 10.1097/MD.0000000000021737

**Published:** 2020-08-21

**Authors:** Bon Sung Koo, Seung Hyeon Lee, So Jeong Lee, Woo Hyun Jung, Yang Hoon Chung, Joon Ho Lee, Sung Hwan Cho, Sang Hyun Kim

**Affiliations:** Department of Anesthesiology and Pain Medicine, Soonchunhyang University Bucheon Hospital, Soonchunhyang University College of Medicine, 170, Jomaru-ro, Bucheon-si, Gyeonggi-do, Republic of Korea.

**Keywords:** cystic adenomatoid malformation of the lung, one-lung ventilation, pediatrics, video-assisted thoracic surgery

## Abstract

**Rationale:**

One-lung ventilation (OLV) is essential for adequate visualization and exposure of the surgical site via a videoscopic approach. Although many instruments facilitating OLV are available, the choice is limited in pediatric patients.

**Patient concerns:**

A 4-year-old female (weight: 18.6 kg, height: 100 cm) was admitted via our pediatric outpatient clinic because of recurrent hemoptysis, 2 weeks in duration. She had no medical or surgical history.

**Diagnosis:**

Contrast-enhanced computed tomography (CT) revealed a 4.5-cm-diameter mass in the left, lower lung lobe. She was diagnosed with a congenital pulmonary airway malformation (CPAM).

**Interventions:**

She was scheduled for emergency lobectomy via video-assisted thoracoscopic surgery (VATS). To ensure successful VATS, OLV was essential. As our hospital lacked a small-diameter fiberoptic bronchoscope and a proper bronchial blocker, we decided to use single-lumen tube (SLT) with adult fiberoptic bronchoscope.

**Outcomes:**

We performed successful bronchoscopic-guided OLV using a SLT. We aligned the tube to the right upper lobar bronchus and Murphy eye to prevent obstruction of the right upper lobe bronchus. At the end of surgery, the endotracheal tube lumen had been narrowed by blood clots, we decided to exchange the tracheal tube. The tube was immediately exchanged. After re-intubation, the pulse oximetry (SpO_2_) then gradually increased.

**Lessons:**

Appropriate preparation and careful management should be considered to perform OLV in pediatric patients without significant complications.

## 1 Introduction

A congenital pulmonary airway malformation (CPAM), previously termed a congenital cystic adenomatoid malformation, is the most common lower respiratory tract malformation. The incidence ranges from 1:10,000 to 1:35,000, regardless of side, sex, or race.^[1]^ CPAM features a hamartomatous proliferation of the terminal respiratory bronchioles to form a multicystic lung mass. The disease may reflect pulmonary developmental arrest or malformation, but the precise etiology remains unknown.^[2]^ The clinical manifestations include infection, malignancy, spontaneous pneumothorax, and dyspnea.^[3]^ The recommended management of both symptomatic and asymptomatic CPAM is resection via video-assisted thoracoscopic surgery (VATS), which is associated with a shorter hospital day, a smaller scar, and less pain than open thoracotomy.^[4]^ VATS has thus become popular. To ensure successful VATS, one-lung ventilation (OLV) is essential to afford adequate visualization and exposure of the surgical site.^[5]^ OLV is sometimes difficult in children because their anatomy differs from that of adults. In addition, the airway is smaller and no small double-lumen tube (DLT) or bronchoscope is available. The preferred intubation method for children younger than 8 years features the use of a single-lumen tube (SLT) and a bronchial blocker. But, problem include failure to achieve an adequate seal of bronchus.^[6]^

Here, we present the case of a 4-year-old child with CPAM who underwent left lower lobe lobectomy via VATS. Patient’ parent has provided informed consent for publication of the case. As our hospital lacked a small-diameter fiberoptic bronchoscope and a proper bronchial blocker, we decided to use SLT with adult fiberoptic bronchoscope.

## 2 Case report

**Figure 1:**
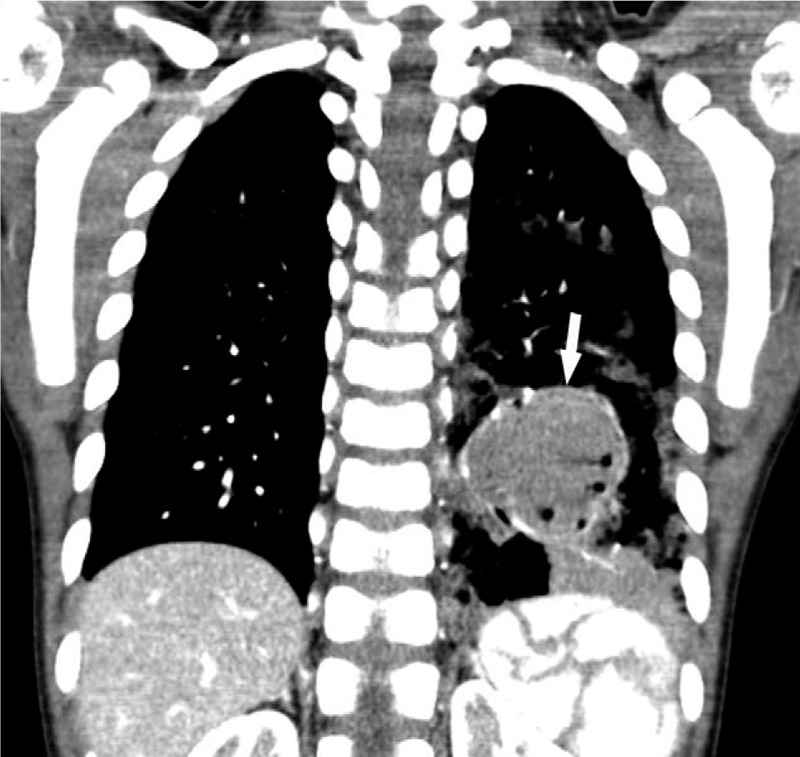
Contrast-enhanced chest computed tomography (CT). A 4.5 cm × 3.4 cm sized cystic pulmonary airway malformation is seen in left lower lobe (arrow).

**Figure 2:**
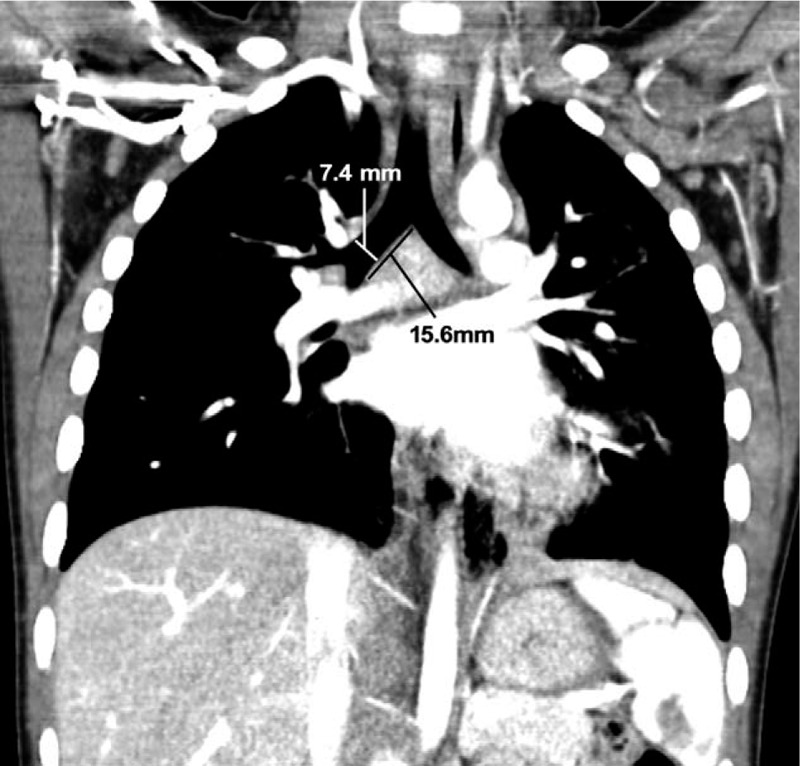
Contrast-enhanced chest computed tomography (CT). The largest diameter of right main bronchus is 7.4 mm. Length from carina to right upper lobar bronchus is 15.6 mm.

A 4-year-old female (weight: 18.6 kg, height: 100 cm) was admitted via our pediatric outpatient clinic because of recurrent hemoptysis, 2 weeks in duration. She had no medical or surgical history. Contrast-enhanced computed tomography (CT) revealed a 4.5-cm-diameter CPAM in the left, lower lung lobe (Fig. 1). She was scheduled for emergency lobectomy. In the coronal reformatted images, the largest diameter of the right main bronchus was 7.4 mm and the distance from the carina to the right, upper lobar bronchus was 15.6 mm (Fig. 2). Initially, we planned to use an SLT 5.0 mm in inner diameter (ID) and 6.9 mm in outer diameter (OD), and a 4-Fr Fogarty catheter as a bronchial blocker. However, during simulation, we found that the bronchoscope and catheter could not simultaneously pass through the lumen of the tracheal tube. An SLT of ID 5.5 mm (OD 7.5 mm) was too large and an SLT of 4.5 mm ID is very difficult to insert and renders bronchoscopy problematic. Our best available choice was an SLT of ID 5.0 mm lacking a bronchial blocker.

**Figure 3:**
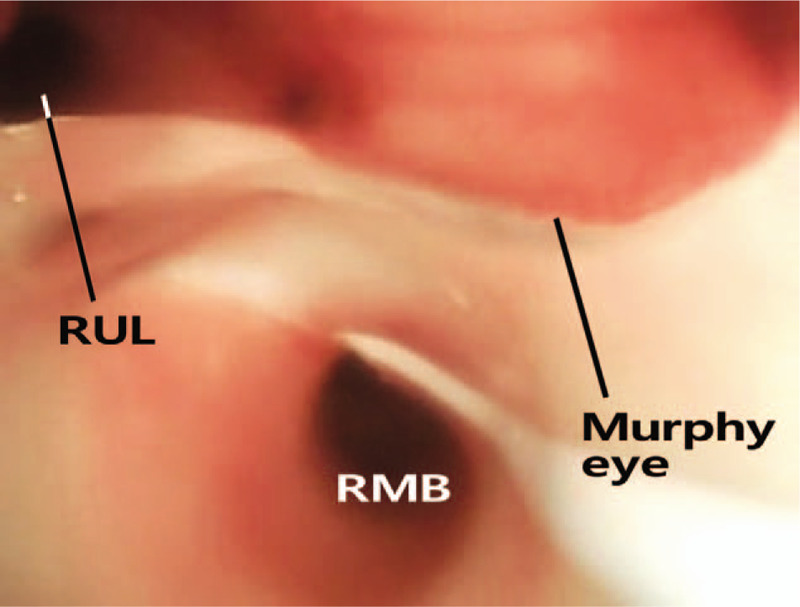
Right upper lobar bronchus (RUL) is connected via Murphy eye. RUL = right upper lobar bronchus, RMB = right main bronchus.

Upon arrival in the operating room, three-lead electrocardiography, pulse oximetry (SpO_2_), and non-invasive blood pressure were monitored. The baseline blood pressure and heart rate were 99/66 mm Hg and 112 beats per minute, respectively. Anesthesia was induced with 5 mg/kg thiopental and 1 mcg/kg remifentanil. Rocuronium 0.5 mg/kg served as the neuromuscular blocker. Sevoflurane and remifentanil were used as maintenance agents. The SLT was intubated videoscopically (McGrath instrument; Medtronic, Minnesota). After the tip of the endotracheal tube passed the vocal cords, we turned the SLT slightly clockwise and advanced it until breath sounds were heard only on the right side. The SLT was fixed 19 cm from the incisors and the cuff inflated with 0.3 mL of air. Next, using a 4.0 × 65 flexible video endoscope (Karl Storz, Germany), we aligned the tube to the right upper lobar bronchus and Murphy eye to prevent obstruction of the right upper lobe bronchus (Fig. 3). All right lung fields (including the apex) exhibited clear breathing sounds. The fraction of inspired oxygen (FiO_2_) was set to 0.6 with saturation at a pulse oximetric level of 100%. At that time, her arterial blood gas analysis revealed a pH of 7.36, PCO_2_ of 40 mm Hg, PO_2_ of 130 mm Hg, bicarbonate level of 21.9 mmol/L.

After induction, a 22G radial artery cannula was placed for monitoring of arterial blood pressure and the right internal jugular vein was accessed with a 5-Fr central catheter. During operation, OLV was successful and the vital signs remained stable. Using a video-assisted thoracoscopic approach, the major vessels and bronchus were dissected, a 4 × 60-mm endoscopic stapler (Endo GIA; Covidien, Ireland) was used to divide and seal the vessels and bronchi.

At the end of surgery, we sought to re-inflate the collapsed lung. After withdrawal of the tube to a depth of 15 cm, both lungs became well-inflated in the absence of bleeding and air leakage. However, when a chest tube was inserted, the SpO_2_ gradually decreased. The peak pressure increased from 15 to 30 cm H_2_O, and the capnographic waveform changed to an obstructive pattern. First, we checked for bending or blockage of the breathing circuit. Next, we immediately increased the FiO_2_ to 0.8 and used the bronchoscope to explore the tracheal tube. The lumen had been narrowed by blood clots, and we sought to remove these using bronchoscopic suction, but failed. As it was still about half an hour to end operation, we decided to exchange the tracheal tube with an endotracheal tube of the same size. The tube was immediately exchanged. After re-intubation, the SpO_2_ then gradually increased and the peak inspiration pressure fell to 14 cm H_2_O at a tidal volume of 150 mL. The lowest SpO_2_ attained was 93% and the duration of desaturation was about 20 minutes. After all surgical procedures were complete, anesthetic agents were withdrawn and neostigmine 1 mg and glycopyrrolate 0.2 mg administered as reversal agents. Extubation was performed and the patient was transferred to the intensive care unit without any complications. She was discharged on postoperative day 4.

## 3 Discussion

Over the past few decades, advances in surgical instrumentation have increased the use of VATS in both adults and children. Although VATS can be performed using retractors with both lungs ventilated,^[7]^ lung isolation is commonly used because of the ease of surgical exposure, good access to the target, and prevention of contamination caused by hemorrhage or infection. The most common method of adult OLV features the use of a DLT. As the airways of young children are too small to permit use of a DLT, other OLV techniques are employed. Single-lumen endotracheal tubes, balloon-tipped bronchial blockers (e.g., Fogarty catheters), and Univent tubes can be placed in small children and infants.

When planning pediatric lung isolation, the “ABCD” considerations: anatomy, bronchoscopy, chest image, and varying diameter of airway with age, are in play.^[8]^ The anesthesiologist must have proficient knowledge of tracheobronchial anatomy in order to optimally place lung isolation devices and troubleshoot problems using fiberoptic bronchoscopy.^[8]^ In addition, as part of the preoperative assessment of the patient, the anesthesiologist should always look at all available chest imaging, x-ray, or CT.^[8]^

A DLT allows simultaneous suctioning and oxygenation of both the operative and non-operative lungs and visualization of the operative lung. However, the smallest DLT is 26-Fr. This DLT is appropriate only for children at least 8 years of age, 30 kg in weight, and 130 cm in height. Therefore, it was not suitable for our case (4 years of age, 18.6 kg in weight, and 100 cm in height). OLV using an SLT renders it necessary to insert the tube into the main bronchus of the operative side. This is simple and rapid. However, this approach may fail if the operative lung collapses, does not protect the airway from contamination, and does not permit suctioning of the operative lung.^[8]^ There are 3 problems with this technique: suction cannot be applied to the operative lung; hypoxemia may result from obstruction of the upper lobe bronchus by the cuff of the endotracheal tube; oxygen and continuous positive airway pressure cannot be administered to the operative lung.^[9]^

A bronchial blocker can be inserted either coaxially (inside the tube) or in parallel (outside the tube). Such techniques completely block the airway. However, a bronchial blocker may damage the airway mucosa if the high-pressure low-volume cuff becomes dislodged from the bronchus.^[8]^ Also, the operative lung cannot be suctioned or placed under continuous positive airway pressure. The Univent tube combines a conventional tube with a bronchial blocker and can be used to treat young children who cannot be treated employing a conventional DLT, but not those aged <6 years.^[7]^ As our patient was 4 years old, we initially planned to use a conventional endotracheal tube and a coaxial Fogarty catheter as the bronchial blocker. On preoperative airway evaluation, we discovered that only a 5-mm-diameter SLT could be employed. Unfortunately, we had only a 4-mm-diameter bronchoscope, which could not pass through the lumen of the tracheal tube if a Fogarty catheter was also present. Our remaining options were insertion of a parallel bronchial blocker (thus, outside the SLT) and endobronchial intubation using a conventional SLT. We decided to intubate with an SLT only because parallel placement of a bronchial blocker was associated with risks of airway damage, balloon misplacement, and hypoxemia.

We chose the size of the SLT by reference to chest CT images. We measured the diameter of the right main bronchus, and the distances from the vocal cords to the carina and from the carina to the right, upper lobar bronchus. We confirmed that no airway variation was present. This reduced the risk of endotracheal tube malpositioning, and the intubation and confirmation times.^[10]^

Intubation of the right bronchus was successful, we used a fiberoptic bronchoscope to adjust tube depth and angle, aligning the tube with the right upper lobar bronchus and Murphy eye. In general, when using SLT to perform OLV, anesthesiologists should pay close attention to obstruction of the right upper bronchus due to cuff. In this case, we aligned the tube to the right upper lobar bronchus and Murphy eye to prevent obstruction of the right upper lobe bronchus. This allowed us to ventilate the right upper lobe. Surgery was proceeding well and the vital signs were stable during lobectomy. At the end of surgery, re-inflation of the operative lung was required and we withdrew the tube from the bronchus to the trachea. At that time, the SpO_2_ then fell and the peak inspiration pressure increased. High peak inspiration pressure can be caused by various factors such as respiratory disease and position, operation type, and problems with the ventilator or airway equipment.^[11]^ Among possible problems with endotracheal tube, endotracheal tube can be obstructed by mucoid secretion, blood clot, or kinking/bending. In this current case, after checking the breathing circuit, we immediately explored the airway bronchoscopically; the tube lumen had been narrowed by blood clots. Perhaps, it was considered that a hemorrhage or debris from the operative lung was regurgitated through the tube during the reinflation of the both lung. Exchanging the endotracheal tube was immediately performed. Since then, there were no other special problems.

In conclusion, we used an SLT to perform OLV successfully using limited instrumentation. Even though the desaturation of short duration arose, rapid recognition and management prevented more serious complications. Appropriate preparation and careful management should be considered to perform OLV in pediatric patients without significant complications.

## Acknowledgments

The English in this document has been checked by at least two professional editors, both native speakers of English. For a certificate, please see: http://www.textcheck.com/certificate/1y5fb9

## Author contributions

**Data curation:** Yang Hoon Chung.

**Resources:** Seung Hyeon Lee, Sung Hwan Cho.

**Software:** So Jeong Lee.

**Supervision:** Sang Hyun Kim.

**Visualization:** Joon Ho Lee.

**Writing – original draft:** Woo Hyun Jung.
